# ChIP-Seq Analysis of AtfA Interactions in *Aspergillus flavus* Reveals Its Involvement in Aflatoxin Metabolism and Virulence Under Oxidative Stress

**DOI:** 10.3390/ijms252212213

**Published:** 2024-11-14

**Authors:** Shurui Peng, Liangbin Hu, Wei Ge, Jiakun Deng, Lishan Yao, Hongbo Li, Dan Xu, Haizhen Mo

**Affiliations:** School of Food Science and Engineering, Shaanxi University of Science and Technology, Xi’an 710021, China; pshurui@sust.edu.cn (S.P.); hulb@sust.edu.cn (L.H.); weige@sust.edu.cn (W.G.); dengjiakun@sust.edu.cn (J.D.); lisa4643@sust.edu.cn (L.Y.); hongbo715@163.com (H.L.)

**Keywords:** *Aspergillus flavus*, transcription factor AtfA, oxidative stress, epigenetic mechanism, pathogenicity, AFB_1_ biosynthesis

## Abstract

The risk of *Aspergillus flavus* contamination is expanding with global warming. Targeting the pathogenicity of *A. flavus* at its source and diminishing its colonization within the host may be a potential control strategy. Oxidative stress transcription factor AtfA plays a pivotal role in *A. flavus* pathogenicity by combating reactive oxygen species (ROS) generated by host immune cells. This study employed chromatin immunoprecipitation sequencing to elucidate the binding sites and epigenetic mechanisms of AtfA under oxidative stress. Among the total 1022 identified potential AtfA-binding peaks, a 10-bp region predominated by 5′-DRTGTTGCAA-3′, which is highly similar to the AP-1 binding motif was predicted. The significantly regulated genes exhibited a variety of biological functions, including regulation of filamentous growth, response to extracellular stimulus, and regulation of gene expression. Moreover, AtfA indirectly influenced these processes via the MAPK signaling pathway, carbon metabolism, and fatty acid metabolism in response to oxidative stress. The absence of *atfA* contributed to the decrease in the growth and development, sporulation, AFB_1_ biosynthesis, and invasion ability of *A. flavus* under oxidative stress. These findings suggest that AtfA is critical to overcome oxidative stress induced by the host immune cells during the infection, providing a novel target for early prevention of *A. flavus* contamination.

## 1. Introduction

*Aspergillus flavus* is an opportunistic pathogenic fungus widely scattered across the world [1]. It stands as a major aflatoxigenic species known to frequently infects peanuts, maize, and cottonseed, and produces detrimental aflatoxins with high carcinogenicity, giving rise to an enormous threat to agricultural economy and human health [2,3]. The infection of *A. flavus* has become an increasing problem with ongoing global warming, taking on a new urgency to control *A. flavus* colonization [4,5].

Reactive oxygen species (ROS) burst is an important signal of hypersensitive response in plants to defend against the pathogen [6,7]. Excessive ROS can subject oxidative stress to *A. flavus* when it infects grains. Based on this condition, fungal cells utilize diverse signal transduction mechanisms to perceive and respond to high external ROS levels [8]. These conserved signaling pathways incorporate a phosphorylation relay system module—the coupled kinase/mitogen-activated protein kinase (SAPK/MAPK) pathway—for transmitting oxidative stress signals, thereby regulating a series of specific oxidative stress-related transcription factors [9,10,11].

The bZIP (basic leucine zipper) transcription factors play a crucial role in oxidative stress-responsive transcriptional regulation in collaboration with the MAPK cascade [12,13]. These proteins can bind DNA sequences to modulate the expression of genes involved in development, secondary metabolism, and virulence in responding to oxidative stress. In filamentous fungi, more than 10 bZIP TFs have been identified to be the key factors mediating oxidative stress, including AtfA, AtfB, AP-1, MeaB, FlbB, RsmA, NapA, JlbA, and so on [9,14]. Among these, AtfA is the common member of the CREB/ATF family that binds CRE sites (5′-TG/TACGTC/AA-3′). Piperine exposure contributed to a reduction in aflatoxin B_1_(AFB_1_) and a significant expression of AtfA in *A. flavus* [15]. Our previous study demonstrated that epigallocatechin gallate (EGCG) could inhibit the production of AFB_1_ and facilitate a significant decrease in the expression of AtfA [16]. Recent investigations have highlighted the multifaceted roles of AtfA in growth, conidiation, aflatoxin biosynthesis, and oxidative stress response, and the *atfA* deletion strain exhibited heightened sensitivity to oxidative stress [17,18]. Although AtfA has been characterized to respond to oxidative stress in *A. flavus*, the binding sites and epigenetic mechanisms of the transcription factor AtfA under oxidative stress remain to be studied. ChIP-seq (chromatin immunoprecipitation followed by sequencing) is considered as an indispensable technique to uncover the target genes and biological mechanism regulated by the transcription factor [19]. In our present study, the binding motif and biological processes regulated by AtfA in response to oxidative stress were investigated by ChIP-seq, and the epigenetic mechanisms were further verified by exposing the ∆*atfA* strain to oxidative stress.

## 2. Results

### 2.1. Bioinformatics Analysis

**Figure 1:**
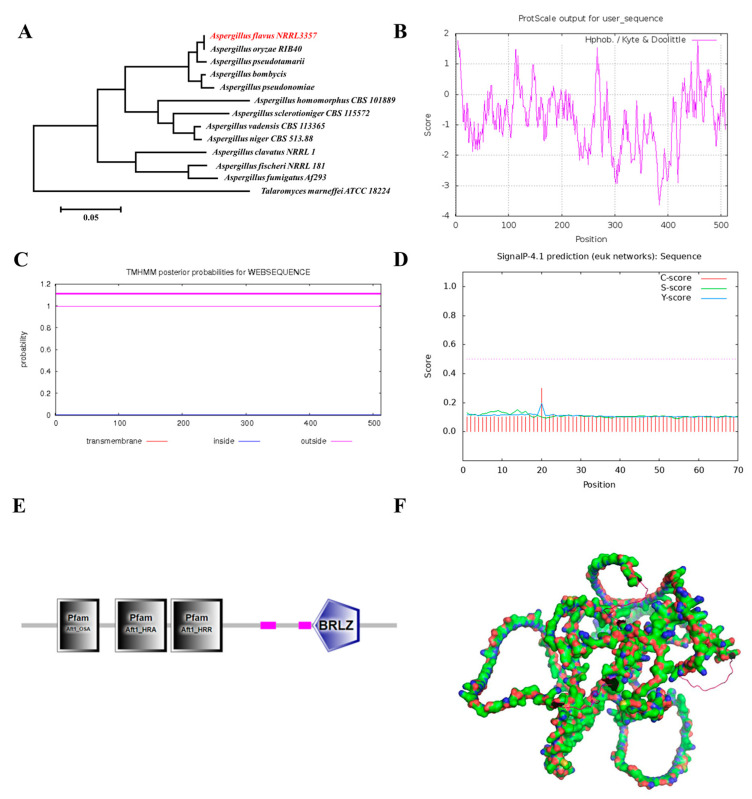
Bioinformatics analysis of AtfA. (**A**) Phylogenetic tree analysis of AtfA in different species. (**B**) The hydropathicity/hydrophobicity prediction of AtfA. (**C**,**D**) The transmembrane domain and signal peptide were analyzed by TMHMM Server v. 2.0 and SignalP-4.1, respectively. (**E**) Domain analysis of AtfA. (**F**) Three-dimensional structure of AtfA was obtained by AlphaFold2 and visualized by PyMOL 1.5.0.3.

To characterize the ortholog of AtfA, a phylogenetic analysis of AtfAs genes cluster search in NCBI (National Center for Biotechnology Information) blast was performed, and the results revealed that AtfA was evolutionarily conserved in the *Aspergillus* species, with AtfA in *A. flavus* sharing the highest similarity with *A. oryzae* (Figure 1A). The lower homology was observed in *Talaromyces marneffei* (68.1%). AtfA was predicted to be a hydrophilic protein; all regions interacting with water are shown in Figure 1B. Through TMHMM Server v. 2.0 and Signal P 4.1 searching, we identified that there was no transmembrane domain and signal peptide in AtfA (Figure 1C,D). Domain analysis of AtfA showed that there was a highly conserved bZIP domain (Figure 1E). Additionally, the three-dimensional structure of AtfA was developed using AlphaFold (Figure 1F). The complex structure specified the potentially important biological functions of AtfA.

### 2.2. Preparation of Polyclonal Antibody

**Figure 2:**
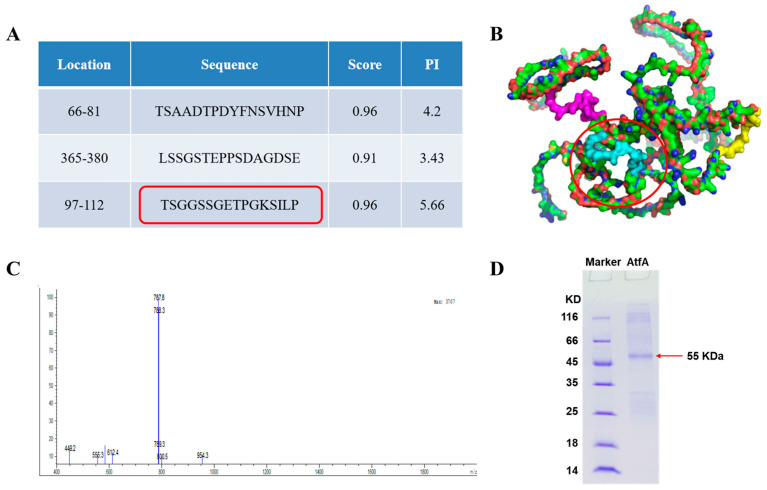
Synthetic peptides and preparation of the AtfA antibody. (**A**) Prediction of antigenic determinant by ABC pred. Three peptides with higher scores were screened. The regions encircled in red was the chosen peptide. (**B**) Three peptides were visualized on three-dimensional structure. The regions encircled in red depict the visualization of the chosen peptide on three-dimensional structure. (**C**) MS analysis of aim peptide. (**D**) SDS-PAGE analysis of antibody purification. The anti-AtfA heavy chain is represented as 55 KDa.

A polyclonal antibody was produced by using a synthesized polypeptide. Through ABC pred searching, three peptides with higher scores were screened. Following a comprehensive analysis combining bioinformatics analysis and visual analysis of the three peptides in three-dimensional structure, the 17-aa C-TSGGSSGETPGKSILP peptide was selected and synthesized (Figure 2A,B). After purification and identification by reverse-phase, high-performance liquid chromatography (RL-HPLC) and mass spectrometry (MS), the polypeptide with a molecular weight of 1577.74 was obtained, which was consistent with the theoretical value predicted by bioinformatics (Figure 2C). Polyclonal antibodies with a concentration of 0.7 mg/mL were produced by immunizing rabbits, and the titer was around 512,000 (Figure 2D, Table S2).

### 2.3. ChIP-Seq Analysis Identifies a Set of AtfA Target Genes in Response to Oxidative Stress

**Figure 3:**
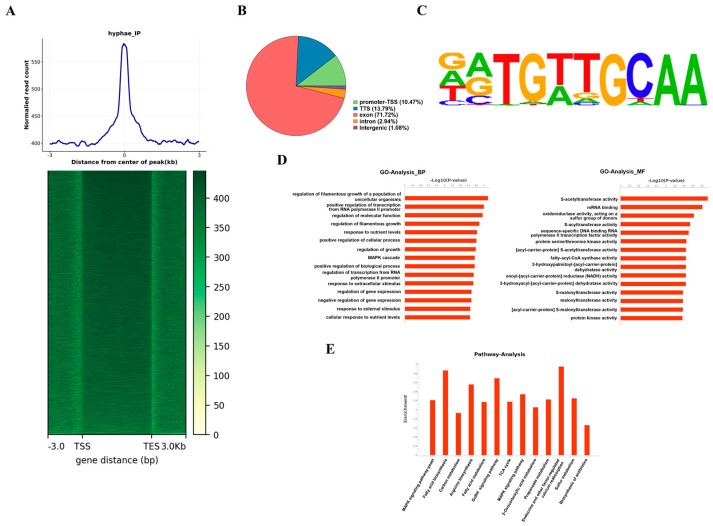
ChIP-seq analysis of AtfA in response to oxidative stress. (**A**) Heatmap of read distributions across the gene. (**B**) ChIP-seq peaks distribution. (**C**) AtfA-binding motif as identified using Homer. (**D**) GO analysis of matching DEGs. (**E**) KEGG analysis of matching DEGs.

To comprehensively investigate the potential regulatory mechanisms of AtfA under oxidation stress, ChIP-seq analysis using AtfA antibody exposure to H_2_O_2_ was conducted. Ultimately, a total of 1022 peaks for AtfA-binding events were identified in response to oxidative stress. Among them, 25% of the peaks were located 3 kb upstream (promoter region) of the translational start sites, with such peaks exhibiting a high signal density (Figure 3A,B). The predicted AtfA-binding motifs were matched with HOMOR. The motif sequences are displayed in Figure 3C, which shows a 10 bp region predominated by 5′-DRTGTTGCAA-3′. GO (Gene Ontology) analysis, which was performed to elucidate the biological function of AtfA target genes. For molecular function, the most enriched GO terms included S-acetyltransferase activity, mRNA binding, oxidoreductase activity, protein serine/threonine kinase activity, malonyltransferase activity, etc. (Figure 3D). For the biological process, AtfA significantly bound to genes related to the regulation of filamentous growth, MAPK cascade, response to extracellular stimulus, regulation of gene expression, response to nutrient levels, positive regulation of transcription from RNA polymerase II promoter, regulation of molecular function, etc. (Figure 3D). Moreover, KEGG (Kyoto Encyclopedia of Genes and Genomes) enrichment results revealed that AtfA target genes were mostly enriched in the MAPK signaling pathway, fatty acid biosynthesis, carbon metabolism, fatty acid metabolism, TCA cycle, and so on (Figure 3E). These results collectively indicated that annotated AtfA-targeted genes were associated with signal transmission, development, secondary metabolism, and pathogenicity in response to oxidative stress.

### 2.4. Construction of AtfA Deletion

**Figure 4:**
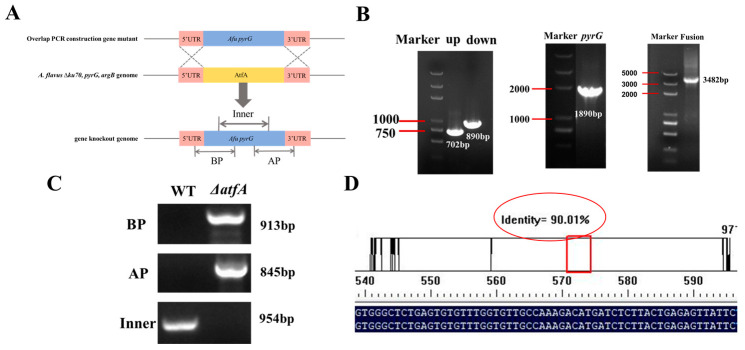
Construction of the *atfA* deletion mutant in *A. flavus*. (**A**) The schematic diagram of *atfA* deletion. (**B**) The three amplified fragments of upstream—*pyrG*—downstream amplified by PCR. (**C**) The construed ∆*atfA* strain was validated by diagnostic PCR with genomic DNA as the template. (**D**) Validation of ∆*atfA* strain by sequencing. The number encircled in red circle represented the comparison of fragment homology in ∆*atfA* with the designed splicing sequence.

To further validate the biological functions of AtfA in the response of *A. flavus* against oxidative stress, the *atfA* knockout strain was constructed through homologous recombination. The schematic diagram is displayed in Figure 4A. Three amplified fragments were fused into a fragment of upstream—*pyrG*—downstream (Figure 4B). Afterward, the deletion of AtfA was validated by PCR. The results showed that the BP and AP fragment could only be amplified from the ∆*atfA* strain, while the inner existing in the WT strain could not be amplified from the ∆*atfA* strain (Figure 4C). These results indicated that *atfA* had been knocked from the ∆*ku70, argB,* and *pyrG^−^* strains. The mutant was further verified by sequencing, and the results showed that the segments were in line with the designed scheme (Figure 4D), which confirmed that the ∆*atfA* knockout strain had been successfully constructed.

### 2.5. AtfA Is Essential for Fungal Growth and Conidial Development Under Oxidative Stress

**Figure 5:**
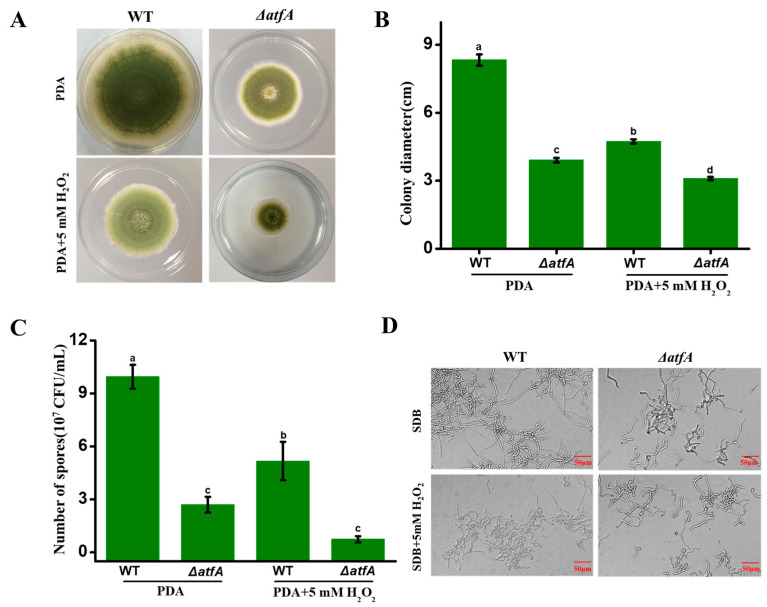
The role of AtfA in the fungal growth and conidial development of *A. flavus* in response to oxidative stress. (**A**) The colony phenotype of WT and ∆*atfA* strains point-inoculated on PDA with 5 mM H_2_O_2_. (**B**) Quantitative analysis of colony diameter shown in (**A**). (**C**) Quantitative analysis of conidia number colony diameter shown in (**A**). (**D**) The conidia germination of the WT and ∆*atfA* strains inoculated in SDB with 5 mM H_2_O_2_. Different letters within the same column indicate significant differences (*p* < 0.05).

In view of the significant enrichment of AtfA-targeted genes in the regulation of filamentous growth, we speculated that AtfA might be involved in the fungal development under oxidative stress. To test this hypothesis, the growth of WT and the ∆*atfA* strain grown on PDA (potato dextrose agar) with 5 mM H_2_O_2_ were measured. As shown in Figure 5A, the ∆*atfA* strain colony was significantly decreased compared to the WT strain on PDA medium. With the addition of H_2_O_2_, both the WT and ∆*atfA* strains displayed a smaller diameter (Figure 5B). Moreover, the ∆*atfA* strain exhibited more sensitivity to oxidative stress. These findings suggested that AtfA, functioning as a transcription factor in response to oxidative stress, was essential for *A. flavus* growth.

Furthermore, the conidia were collected and counted to evaluate the function of AtfA in spore formation under oxidative stress. After 5 days of growth on PDA, conidia production of the ∆*atfA* strain was obviously less than that in the WT strain (Figure 5C), which indicated AtfA played an important role in the conidia production of *A. flavus*. Additionally, the inhibition rate of conidia production in the ∆*atfA* strain was significantly higher than that in the WT strain under the oxidative stress. It reflected that AtfA participated in the conidia production against H_2_O_2_-mediated oxidative stress.

To assess the role of AtfA in conidia germination under oxidative stress, the spores of the WT and ∆*atfA* strains were inoculated on SDB (Sabouraud dextrose broth) medium with 5 mM H_2_O_2_. Unlike in the WT strain, the conidia germination rate in the ∆*atfA* strain obviously slowed, implying that AtfA might be critical for conidia germination in *A. flavus* (Figure 5D). Compared to normal conditions, the conidia germination of the WT strain was significantly inhibited under oxidative stress. Whereas, the absence of AtfA exhibited little impact on conidia germination, similar to that observed under normal conditions. Taken together, the results indicated that AtfA might play an important regulatory role in conidia germination under oxidative stress.

### 2.6. AtfA is Involved in AFB_1_ Biosynthesis Under Oxidative Stress

**Figure 6:**
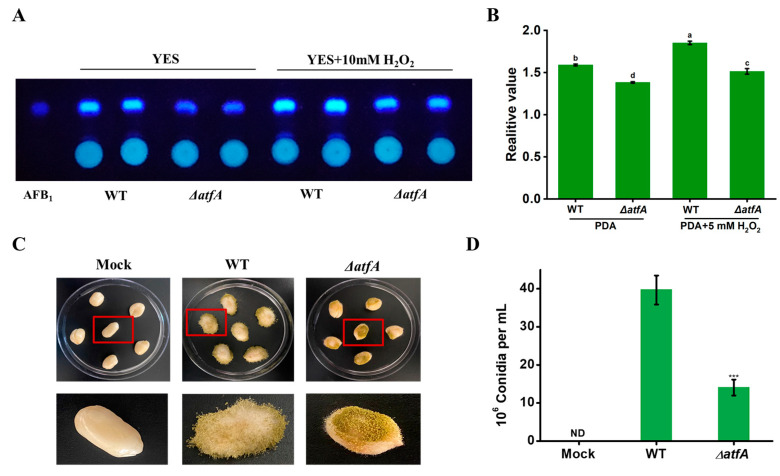
The function of AtfA in the AFB1 biosynthesis and virulence of *A. flavus* under oxidative stress. (**A**) TCL analysis of the production of AFB_1_ in the above *A. flavus* strains in liquid YES media with 5 mM H_2_O_2_. (**B**) Relative quantification of AFB_1_ production by Image J 1.54f according to the result shown in Figure (**A**). (**C**) The colonization of WT and ∆*atfA* on peanut seeds. The samples in the red box were displayed separately below. (**D**) The conidia number washed from the infected peanut seeds. ***: *p* < 0.001. Different letters within the same column indicate significant differences (*p* < 0.05).

Oxidative stress is an important element of AFB_1_ biosynthesis [20]. To further confirm the function of AtfA in the regulation of secondary metabolism under oxidative stress, strains were inoculated on YES (yeast extract sucrose) medium supplemented with 5 mM H_2_O_2_. TLC (thin-layer chromatography) analysis revealed a significant reduction in AFB_1_ production in the *atfA* deletion strain compared to the WT strain in YES medium (Figure 6A,B). Furthermore, the AFB1 production in the WT strain had a remarkable increase relative to the ∆atfA strain under oxidative stress. These results thus indicated that AtfA was critical for the biosynthesis of AFB_1_ in resistance to oxidative stress.

### 2.7. AtfA Influences the Pathogenicity of A. flavus

*A. flavus* frequently contaminates grain and oils crops, leading to a great economic loss. The impact of AtfA on the infection ability to peanuts was characterized. Results demonstrated that a large number of conidia were attached to the surface of peanuts infected by WT strain, while the conidia observed on peanuts obviously decreased after inoculated with ∆*atfA* strain (Figure 6C). Conidia on the surface of peanuts were then harvested and quantified, the amount of conidia on peanuts inoculated with the ∆*atfA* strain was decreased by nearly 70%. All these data indicated that AtfA had a critical role in the infection capability of *A. flavus* (Figure 6D).

## 3. Discussion

The bZIP transcription factors are well known to be involved in various biological processes such as development, stress response, virulence, and secondary metabolism in filamentous fungi. In our pervious study, we proposed a hypothetical mechanism in which ROS might activate the transcription factor AtfA via the MAPK signaling pathway, and the decreased expression of genes related to AFB_1_ biosynthesis might be the downstream response to AtfA. Recent studies have shown that AtfA plays a crucial role in the mycelium growth, sclerotia formation, conidia formation, aflatoxin biosynthesis, and defense against oxidative stress, and the deletion of *atfA* leads to an enhanced sensitivity to oxidative stress [17,18]. Yet little is known about AtfA-binding target genes and the mechanism regulated by AtfA under oxidative stress.

High homology of AtfA was discovered among *Aspergillus* spp., especially *Aspergillus oryzae*, which suggested that AtfA in *A. flavus* may play similar roles in the biological processes as observed in other *Aspergillus* spp. under oxidative stress. As a member of the ATF/CREB family of proteins in *A. oryzae*, AtfA could regulate some genes putatively involved in oxidative stress resistance that are not regulated by AtfB, controlling germination and stress tolerance of conidia [21]. Similarly, AtfA has been shown to regulate several stress-protection-related genes such as *catA*, *dprA*, *scf1*, and *conJ* at the conidiation stage via the HOG MAPK pathway in *Aspergillus fumigatus* [22]. Additionally, the ATF/CREB transcription factor AtfA has been demonstrated to play critical roles in oxidative stress responses during growth and development in *Aspergillus nidulans* [23]. Hence, AtfA may be the ATF/CREB transcription factor regulating stress-protection-related genes at the conidiation and development stage.

ChIP-seq was employed to identify the genes directly bound by AtfA in response to oxidative stress. Among the 1022 identified peaks, only 25% of the genes were located in the upstream 3 kb region, proposing a model in which AtfA-mediated regulation controls downstream gene cascades via direct and indirect interactions in response to oxidative stress. Our study identified the consensus motif 5′-DRTGTTGCAA-3′ as the binding motif for AtfA, which is highly similar to the AP-1 binding motif. It was reported that Ap-1 binds to the consensus motif (5′TGAC/GTCA3′, 5′TT/GACTAA3′) of some target genes [24,25]. GO enrichment analysis revealed that these genes were involved in the regulation of filamentous growth, positive regulation of the cellular process, positive regulation of transcription from the RNA polymerase II promoter, response to nutrient levels, and regulation of gene expression. In addition to direct regulation of some genes related to growth, nutrient levels and secondary metabolism, AtfA also indirectly affects biological processes via other metabolic pathways in response to oxidative stress. The KEGG pathway analysis revealed that AtfA target genes were mostly enriched in the MAPK signaling pathway, fatty acid biosynthesis, carbon metabolism, fatty acid metabolism, and the TCA cycle under oxidative stress. As one of the most important phosphorylation pathways, MAPK cascade modules convert extracellular stimuli into cellular responses, including development, signal transduction, and secondary metabolism [26,27]. Carbon metabolism generates acetyl-CoA, a key precursor for AFB_1_ synthesis [28]. Likewise, the TCA cycle, which utilizes acetyl-CoA and fatty acids to produce energy in aerobic organisms, shares a similar starting substrate with AFB_1_ synthesis [29,30]. Therefore, the TCA cycle is intricately linked to AFB_1_ synthesis.

The *atfA* knockout strain was constructed to further validate the biological functions in response to oxidative stress. Deletion of *atfA* contributed to the decrease in the growth, sporulation, AFB_1_ biosynthesis, and invasion ability of *A. flavus* under oxidative stress. Similar oxidative stress phenotypes have also been found in other bZIP transcription factors of *A. flavus*, such as AP-1, AtfB, and RsmA [17,18,31,32]. These findings indicate that oxidative stress transcription factors play a crucial role in the oxidative stress in the development and secondary metabolism of *A. flavus*. It is worth noting that the absence of AtfA had little effect on conidia germination under oxidative stress, indicating that ∆*atfA* is not more sensitive to oxidative stress in conidia germination than that in the normal condition. In *A. nidulans*, the absence of *atfA* increases the stress sensitivity of mutant conidia onto tBOOH-supplemented stress plates [33]. Similarly, the *ΔatfA* mutant is sensitive to H_2_O_2_, leading to inhibition of conidial germination in *A. oryza* [21]. These oxidative stress-sensitive phenotypes have also been discovered in *A. fumigatus*, *Fusarium graminearum*, and *Magnaporthe oryzae* [22,34,35], although the ∆*bcatf1* mutant is not hypersensitive to oxidative stress in *Botrytis cinerea* [36]. This difference may be attributed to the deletion of AtfA, resulting in impaired spores and subsequently a weaker sense of the environment changes.

## 4. Materials and Methods

### 4.1. Strains and Chemicals

The strains *A. flavus* NRRL3357, *A. flavus* NRRL3357∆*ku70*, ∆*argB*, *pyrG*^−^, and *A. flavus* NRRL3357∆*ku70*, ∆*argB* were kindly provided by Prof. Zhumei He (Sun Yat-sen University, Guangzhou, China). These strains were incubated in PDA medium (BDDifco, Franklin, NJ, United States) at 30 °C for 6 d, with appropriate amounts of arginine, uridine, and uracil added to the medium as required. Conidia were harvested in 0.9% caline containing 0.05% Tween-80 and their quantities were determined using a hemocytometer. All other chemical agents were analytical grade. 

### 4.2. Sequence Resources and Bioinformatics Analysis

The protein sequence of AtfA and its homologous sequences were obtained from the NCBI database (http://www.ncbi.nlm.nih.gov) (accessed on 11 November 2024). The hydropathicity/hydrophobicity and transmembrane domain of AtfA were predicted by ProtScale at https://web.expasy.org/protscale/ (accessed on 11 February 2022) and TMHMM Server v. 2.0 at https://services.healthtech.dtu.dk/services/TMHMM-2.0/ (accessed on 11 February 2022), respectively. Signal peptide prediction was performed using SignalP-4.1 (https://services.healthtech.dtu.dk/services/SignalP-4.1/) (accessed on 13 February 2022). Protein domain was analyzed using the website SMART (http://smart.embl-heidelberg.de/) (accessed on 2 March 2022). The AtfA protein of all these 13 species were aligned by ClustalW using MEGA software 11.0.13 to establish the phylogenetic tree. The three-dimensional structure of AtfA was generated via homology modeling using AlphaFold2 on the CoLabFold publicly accessible interface (https://alphafold.com/) (accessed on 11 November 2024) and was visualized by PyMOL 1.5.0.3.

### 4.3. Synthetic Peptides and Preparation of the Antibody of AtfA

The antigenic determinant of AtfA was predicted using the ABC pred website (https://webs.iiitd.edu.in/raghava/abcpred/index.html) (accessed on 7 March 2022). The optimal sequences were selected for peptide synthesis using the solid-phase peptide synthesis method. Purification and analysis of the crude peptides were carried out by RP-HPLC (LC-2010A, Shimadzu, Kyoto, Japan) and MS (Waters ZQ2000, Milford, MA, USA). The RP-HLPC analysis was performed on a Sinochrom ODS-BP C18 (4.6 × 250 mm, 5 μ) column at a flow rate of 1 mL/min; solvent A: 0.1% Trifluoroacetic in 100% acetonitrile; solvent B: 0.1% Trifluoroacetic in 100% water. The MS analysis was performed on a mass spectrometer equipped with a standard ESI source. The instrument was operated in positive ion mode with a voltage of 1.5 kV and a source temperature of 250 °C. The rabbit polyclonal anti-AtfA antibodies were produced by the Zoonbio biotechnology company (Nanjing, China). The titers of purified antibodies were determined by indirect enzyme-linked immunosorbent assay (ELISA). SDS-PAGE was used to assay the purity of the antibody.

### 4.4. Chromatin Immunoprecipitation Sequencing (ChIP-Seq)

Conidia (10^5^ CFU/mL) of *A. flavus* were inoculated into 100 mL SDB medium with 5 mM H_2_O_2_ supplement and shaken at 150 rpm under 30 °C for 24 h. Mycelia were filtered before washing with phosphate-buffered saline (PBS) and frozen with liquid nitrogen. ChIP assays were carried out following the protocol by Boedi et al. with slight modifications [37]. Briefly, mycelia were cross-linked with 1% formaldehyde for about 10 min. Subsequently, the chromatin was extracted and subjected to sonication. After being segmented to 100–500 bp, 10% of the chromatins were immunoprecipitated with specific antibodies at 4 °C for 6 h. Immunoprecipitated DNA were then disposed with RNase A and Proteinase K in turn. Purification of the extracted DNA was performed using the QIAquick PCR Purification Kit (Qiagen, Dusseldorf, Germany). Libraries were produced using NEBNext Ultra II DNA Library Prep Kit for Illumina and sequenced on the Illumina NovaSeq PE150. ChIP-seq reads were trimmed and cleaned using Trimmomatic software Version 0.36 (http://www.usadellab.org/cms/index.php?page=trimmomatic) (accessed on 11 November 2024). Before read mapping, clean reads were obtained from the raw reads by removing the adaptor sequences. The clean reads were then aligned to the *A. flavus* NRRL3357 genome derived from NCBI using BWA-MEM software (https://github.com/lh3/bwa/) (accessed on 11 November 2024). Peak calling was performed in MACS2 software (https://www.megasoftware.net/dload_win_gui) (accessed on 11 November 2024) with a cutoff *p*-value < 0.05. HOMER’s findMotifsGenome.pl tool was applied to predict AtfA-binding motifs and the sequence was compared with the motif databases. Fisher’s exact test was used for GO analysis and KEGG analysis using an FDR (false discovery rate) cutoff of 0.05 [38].

### 4.5. Construction of atfA Deletion Strain

The *ΔatfA* strain generated in this study was following the method of homologous recombination [39]. Briefly, three fragments (upstream and downstream from the *atfA*, *pyrG* gene of *A. fumigatus*) were individually amplified and overlapped by using the primers listed in Table S1. Subsequently, these fragments were ligated together to obtain a fragment of upstream—*pyrG—*downstream. The fused fragment was then transferred into the protoplasts by following the PEG chemo-induction method. The transformants were verified by PCR and sequencing.

### 4.6. Analysis of Fungal Conidia Under Oxidative Stress

To observe the impact of AtfA on the development of *A. flavus* in response to oxidative stress, 10^3^ conidia were point-inoculated on the PDA medium with 5 mM H_2_O_2_ and incubated in darkness at 30 °C. The diameters were measured and conidia were harvested with 0.05% Tween-80 after 5 days inoculation. The numbers of conidia were counted by using a hemocytometer.

### 4.7. Spore Germination Analysis Under Oxidative Stress

To investigate the effect of AtfA on the spore germination of *A. flavus* under oxidative stress, spores (10^6^ CFU/mL) were inoculated into 15 mL SDB medium amended with 5 mM H_2_O_2_ and kept shaking at 150 rpm under 30 °C for 12 h. Spore germination was observed every 2 h and photographed by an inverted fluorescent microscope (IX73P1F, Olympus, Tokyo, Japan).

### 4.8. Determination of AFB_1_ Production Under Oxidative Stress

The role of *atfA* in AFB_1_ production was assessed following the previous methods with slight modification [40]. The conidia suspension was inoculated into 20 mL YES liquid medium containing 5 mM H_2_O_2_ at 30 °C in the dark for 5 days. AFB_1_ production was detected by TLC analysis. Briefly, 10 μL of the aflatoxin extracts were spotted on thin-layer chromatography silica gel plate. The plates were developed in a mobile phase consisting of chloroform: acetone (9:1). The biotransformation products on the plates were visualized using a Four-Purpose UV Analyzer (WFH-203C, Shanghai Chitang Industrial Co., Ltd., Shanghai, China) at a wavelength of 365 nm.

### 4.9. Peanuts Infection Assay

Peanuts with intact skins were selected to assess the biological function of *atfA* in the infection of *A. flavus* on plants [41]. The seeds were washed with 75% ethanol and sterile water, and subsequently inoculated with 10^3^ conidia and cultured at 30 °C for 7 days. The infected seeds were transferred into 50 mL centrifuge tubes containing 10 mL 0.05% Tween-80, followed by vortexing to collect spores and counting for further quantification.

### 4.10. Statistical Analysis

All data were displayed as the mean ± standard deviation (SD) of three independent biological replicates. Statistical analysis was performed with IBM SPSS Statistics 20 using one-way analysis of variance (ANOVA) with Duncan’s test. Statistically significant difference was considered when *p* < 0.05.

## 5. Conclusions

In this study, we systematically explored the binding sites and epigenetic mechanisms of AtfA in *A. flavus* under oxidative stress by ChIP-seq. Our findings revealed that AtfA plays a critical role in the growth and development, sporulation, AFB_1_ biosynthesis, and invasion ability under oxidative stress, providing new insights for preventing and controlling *A. flavus* infection and contamination in the food industry.

## Data Availability

Data will be available on request.

## Supplementary Materials

The following supporting information can be downloaded at: https://www.mdpi.com/article/10.3390/ijms252212213/s1.
