# Supplementary material for: ChIP-Seq Analysis of AtfA Interactions in Aspergillus flavus Reveals Its Involvement in Aflatoxin Metabolism and Virulence Under Oxidative Stress
Source: Int J Mol Sci. 2024 Nov 14;25(22):12213. doi: 10.3390/ijms252212213 (PMC11594458; doi:10.3390/ijms252212213)
Supplement: Supplementary file 1 [file ijms-25-12213-s001.zip › ijms-3312677-supplementary.pdf]

Table S1 Primer sequences

Primer name	Sequence（5'-3'）
AtfA-up-F	CTGTCTCCAAGTCCTTGTTGC
AtfA-up-R	GGGTGAAGAGCATTGTTTGAGGCTTGTTGATGTCCGTCCGTTAT
AtfA-down-F	GCATCAGTGCCTCCTCTCAGACTCTCATTGCGTTTCAGTTT
AtfA-down-R	TAGTTTTATTCACGACCCCTC
pyrG-F	GCCTCAAACAATGCTCTTCACCC
pyrG-R	GTCTGAGAGGAGGCACTGATGC
Check-up-F	ACAGTTGAGGTTGCACTTTAGC
Check-up-R	GTTGAAGTAGCCGAGCAATGAGGT
Check-down-F	AGTGCAGTCGGATGTGTCTTC
Check-down-R	CATGGACTAGGGTTTCAAGATG
AtfA-F	ACCACCTGATTCATCTCATATAC
AtfA-R	CACCTTGCACGTCAGACACGT

Table S2 The test result of antibody purification

Number	Dilution	A450 nm
1	1:500	4.000
2	1:1000	3.998
3	1:2,000	3.967
4	1:4,000	3.912
5	1:8,000	3.875
6	1:16,000	3.097
7	1:32,000	2.262
8	1:64,000	1.437
9	1:128,000	0.797
10	1:256,000	0.508
11	1:512,000	0.293
12	Blank group	0.112
13	Titer:	>512,000

Note: Initial Dilution Ratio: 1:500; The titer is the highest dilution at which the OD of the sample/OD of the blank$\geqslant$2.1.
